# BSE49, a diverse, high-quality benchmark dataset of separation energies of chemical bonds

**DOI:** 10.1038/s41597-021-01088-2

**Published:** 2021-11-23

**Authors:** Viki Kumar Prasad, M. Hossein Khalilian, Alberto Otero-de-la-Roza, Gino A. DiLabio

**Affiliations:** 1 Department of Chemistry, University of British Columbia, Kelowna, British Columbia V1V 1V7 Canada; 2 Departamento de Química Física y Analítica, Facultad de Química, Universidad de Oviedo, MALTA Consolider Team, E-33006 Oviedo, Spain

**Keywords:** Computational chemistry, Quantum chemistry

## Abstract

We present an extensive and diverse dataset of bond separation energies associated with the homolytic cleavage of covalently bonded molecules (A-B) into their corresponding radical fragments (A^.^﻿ and B^.^﻿). Our dataset contains two different classifications of model structures referred to as “*Existing”* (molecules with associated experimental data) and *“Hypothetical”* (molecules with no associated experimental data). In total, the dataset consists of 4502 datapoints (1969 datapoints from the *Existing* and 2533 datapoints from the *Hypothetical* classes). The dataset covers 49 unique X-Y type single bonds (except H-H, H-F, and H-Cl), where X and Y are H, B, C, N, O, F, Si, P, S, and Cl atoms. All the reference data was calculated at the (RO)CBS-QB3 level of theory. The reference bond separation energies are non-relativistic ground-state energy differences and contain no zero-point energy corrections. This new dataset of bond separation energies (BSE49) is presented as a high-quality reference dataset for assessing and developing computational chemistry methods.

## Abstract (summary)

**Table:** 

Measurement(s)	bond separation energies
Technology Type(s)	ab initio quantum chemistry computational method
Factor Type(s)	existing or hypothetical model structure

Machine-accessible metadata file describing the reported data: 10.6084/m9.figshare.16680787

## Background & Summary

Bond dissociation enthalpies (BDEs) are a central property in chemistry that have been studied for decades experimentally and computationally^1–4^. BDEs can be used to estimate the selectivity and reactivity of various molecules with free radicals (like ^·^OH, ^·^OOH, ^·^OR, ^·^OOR, ^·^NO, ^·^NO﻿_2_, etc.) that are generated and transformed during chemical reactions relevant in chemistry and biology^5–10^. In this context, the calculation of BDEs for C-H, O-H, N-H, S-H, O-O, and S-S bonds in biologically relevant systems can help develop an understanding of the efficiency of antioxidants^11–13^. Furthermore, the calculation of BDEs is fundamental to develop a deeper understanding of various enzyme catalytic processes^14–16^ and surface functionalization chemistry^17–19^.

In 2012, Drew and Reynisson employed BDE calculations to predict the major metabolic sites of fifty known drug molecules^20^. Similarly, Andersson and co-workers applied BDE calculations to estimate the sensitivity of various drug candidates toward autoxidation^21^. The application of computed BDEs in these works shows how computational techniques can be incorporated into the risk assessment of drug products and guide further experimentation. Computationally obtained BDEs were also reported in different studies^22–24^, where the C-O and C-C BDEs were calculated for several substituted analogues of lignin, an abundant polymeric organic material and a potential renewable source of biofuels and chemicals^22–24^. The calculated BDEs were used to predict the homolytic dissociation of C-C and C-O bonds under thermal decomposition using model compounds representing the dominant linkages of lignin.

Given the importance of BDEs in many areas of chemistry and, consequently, the need to accurately predict bond energies computationally, a dataset of accurately predicted bond separation energies (BSEs) is developed here using an accurate computational chemistry method. Bond separation energies are a molecular property that can be computed in a straightforward manner in vacuum and provides direct information about the strength of a chemical bond. The BSEs presented in this work are differences between non-relativistic ground-state energies and contain no vibrational energy contributions, no zero-point energies, and no attempt has been made at thermally averaging over molecular conformations. As such, the reported BSEs are not comparable to experimental BDEs, but they serve as an ideal resource for developing and evaluating lower-cost computational chemistry methods used for a wide range of applications in chemistry and biology. Similar datasets to the one proposed in this work are available in the literature, but they tend to be small in terms of the total number of datapoints^25^, lack bond-type diversity^26,27^ or are calculated using less accurate computational chemistry methods compared to the one used in this work^28–30^. To the best of our knowledge, an accurate and extensive dataset of computationally predicted BSEs is not available in the literature. The main reason for this absence is that BSE calculations with high accuracy require computationally expensive methods that tend to scale poorly with system size.

This work addresses the aforementioned gap in the literature by constructing a large dataset (4502 datapoints) of computationally predicted BSEs of 49 unique bond types, all of which are determined with a high-level composite theoretical procedure denoted as (RO)CBS-QB3^31–33^. This approach ensures uniform, high-quality reference data and eliminates the need to collect and verify data gathered from various sources, which may differ substantially in their accuracy. The (RO)CBS-QB3 method is known to produce BDEs of high accuracy^8,33–37^. Therefore, it is suitable for developing a database of BSEs that can be used to test and parametrize low-cost computational methods. One particular target application of our dataset is for the training of cost-effective computational approaches like atom-centered potentials^38–40^ (ACPs) or machine learning potentials^28–30^.

## Methods

### Dataset composition

We present the BSE49 dataset, which comprises a broad range of bond separation energies for 49 unique bond types. The model systems present in the dataset are neutral molecules with X-H, X-F, X-Cl, X-X, and X-Y single bonds, where X and Y are B, C, N, O, Si, P, and S. The number of datapoints and the ranges of bond separation energies associated with each bond type are provided in Table 1. The structures of model systems on which the calculations were performed are divided into *“Existing”* and “*Hypothetical”* classes. The *Existing* type structures were built by selecting molecules with experimental data reported in the *Comprehensive Handbook of Chemical Bond Dissociation Energies*^41^. In contrast, the *Hypothetical* type structures were constructed by functional group substitutions of X-Y single bonds in order to include bond types that were not present in the handbook and to increase the diversity and number of datapoints for each bond type in the dataset. The candidate molecules for both *Existing* and *Hypothetical* subsets were generated using a partially automated computational workflow as described below.

**Table 1:** List of the number of datapoints in the BSE49 dataset and the ranges of bond separation energies associated with each bond type calculated using (RO)CBS-QB3.

Bond type	Datapoints	Range of bond separation energies
B-H	68	77.22–115.14
C-H	395	80.08–141.22
N-H	156	53.05–131.63
O-H	240	68.65–126.75
Si-H	111	74.31–106.06
P-H	118	61.73–87.98
S-H	39	74.80–95.81
B-B	75	47.41–112.40
B-C	83	92.26–142.78
B-N	71	85.50–155.16
B-O	51	100.14–158.50
B-F	82	152.61–177.24
B-Si	84	36.27–110.83
B-P	89	72.64–99.12
B-S	51	84.10–128.28
B-Cl	81	81.86–128.98
C-C	363	64.69–156.08
C-N	98	27.65–122.95
C-O	171	48.31–127.45
C-F	40	103.44–133.45
C-Si	153	36.82–111.67
C-P	85	60.93–115.15
C-S	64	41.42–105.29
C-Cl	129	64.26–113.54
N-N	37	15.64–70.81
N-O	31	22.50–70.80
N-F	36	49.72–83.45
N-Si	64	33.93–122.94
N-P	93	40.82–91.06
N-S	53	24.53–72.61
N-Cl	31	35.89–80.63
O-O	60	21.20–56.42
O-F	90	11.04–51.79
O-Si	144	74.85–144.88
O-P	27	83.10–130.79
O-S	51	46.55–93.05
O-Cl	85	9.38–61.56
F-Si	36	123.92–169.04
F-P	32	99.43–125.94
F-S	99	72.84–107.41
Si-Si	165	34.86–104.94
Si-P	65	60.09–87.04
Si-S	57	62.95–98.18
Si-Cl	102	109.68–123.12
P-P	20	44.37–77.37
P-S	29	67.42–96.01
P-Cl	32	69.91–89.30
S-S	64	37.09–78.33
S-Cl	102	50.44–71.17

The bond separation energy ranges are in kcal/mol.

### Dataset generation

The calculated bond separation energies are defined as the negative of the difference in the ground-state electronic energies for the reaction

$${\rm{A}} \mbox{-} {\rm{B}}\to {\rm{A}}.+{\rm{B}}.$$

where A^.^﻿ and B^.^﻿ represent the two radical fragments formed by homolytically breaking the A-B covalent bond in vacuum. Based on this reaction, the equilibrium geometries of the parent molecules and their respective radical fragments are required to calculate the bond separation energies. The geometries of the parent molecule and the associated radicals were constructed manually for both *Existing* and *Hypothetical* subsets using the *Avogadro*^42^ program. The constructed geometries were then used as starting points for a conformer search. The *CSD conformer generator*^43^ and *FullMonte*^44^ codes were used to generate multiple conformers. The geometry of each conformer was relaxed to the corresponding local minimum using the *Gaussian*^45^ software package. This relaxation was carried out first by using a low-level method, combining the B3LYP^46–51^ density functional and 6-31G*^52,53^ basis set along with the D3^54–56^ dispersion correction scheme using the Becke-Johnson^57^ damping (B3LYP-D3(BJ)/6-31G*). The optimized conformers were ranked using the B3LYP-D3(BJ)/6-31G* relative energies at the local minima. The ten lowest-energy conformers were then re-optimized at the higher-level CAM-B3LYP-D3(BJ)/def2-TZVP level of theory^54–59^. Range-separated functionals like CAM-B3LYP minimize the delocalization error, which could be important in the description of radical species^60^. The lowest-energy conformer obtained in this procedure was used for calculating the bond separation energies using the composite method described below. All calculations employed a default self-consistent field (SCF) convergence criterion of 10^−8^ Hartrees, *ultrafine* integration grid, and a *tight* optimization convergence criteria (maximum force = 1.5 × 10^−5^ Hartrees/Bohr, RMS force = 1 × 10^−5^ Hartrees/Bohr, maximum displacement = 6 × 10^−5^ Bohr, RMS displacement = 4 × 10^−5^ Bohr).

This partially automated workflow produced structures that are not necessarily the global minima. A visual inspection of the structures revealed that about 20% of the conformers generated do not correspond to the global minima, which reflects the difficulty of solving a global optimization problem (finding the most stable conformer) for such a large number of systems reliably. In addition, due to computational constraints, no attempt was made at evaluating the conformational energy landscape and statistically weighting the low-energy conformers associated with each molecule. Therefore, the dataset is not appropriate for direct comparison to bond separation energies obtained by back-correcting experimental BDEs, but it is suitable for testing and training computationally less expensive methods regarding their ability to accurately calculate the energy difference between the chosen conformers of products (A^.^﻿ and B^.^﻿) and reactant (A-B).

The structures obtained from the workflow described above were then used for the final step of reference data calculation, using the composite (RO)CBS-QB3^31–33^ method. The restricted-open-shell^61^ CBS-QB3 or ROCBS-QB3 was employed for the open-shell radical fragments, while restricted closed-shell calculations were performed for the closed-shell parent molecules with CBS-QB3. The composite (RO)CBS-QB3 method approximates energies at the complete-basis-set CCSD(T) level, using a series of computationally lower-cost methods including: (i) geometry optimization followed by vibration frequency calculation using the unrestricted-open-shell^62^ B3LYP/6-311G(2d,d,p) method^46–51,63^, (ii) ROMP2/6-311+G(3d2f,2df,2p) level^63–65^ energy extrapolated to the complete-basis-set limit, (iii) energy calculation at ROMP4(SDQ)/6-31+G(d(f),p) level^63,64,66^, and (iv) energy calculation at ROCCSD(T)/6-31+G† level^63,64,67^ (where 6-31+G† is a modified 6-31+G(d) basis set). Note that the final (RO)CBS-QB3 energy includes additional empirical correction terms described in Reference^33^. Structures were screened to remove any system for which the imaginary frequencies were obtained. The (RO)CBS-QB3 energies for the structures associated with a particular bond breaking reaction were used to obtain the bond separation energies for the dataset.

## Data Records

The reference bond separation energies (in kcal/mol) and coordinates (in Å) of the structures presented in the BSE49 dataset are publicly available free-of-charge from the Figshare^68^ and GitHub (https://github.com/aoterodelaroza/bse49) repositories in the plain-text database file format (DB format) described in Table 2. The atomic coordinates of the model structures are stored in a plain-text XYZ format in the *Geometries* directory. The BSE49 dataset contains one DB format file and three XYZ format files for each bond separation energy. In total, deposited files include 4502 DB format files stored in the *db-BSE49* directory and 13506 XYZ format files stored in their respective *Existing* or *Hypothetical* classification directories. Additional files labelled as *BSE49_Existing.org* and *BSE49_Hypothetical.org* are also provided. These files contain the necessary information about the reference data for all the model systems.

**Table 2:** A description of the DB format file (.db) for an A-B molecule containing N number of atoms with two radical fragments (A^.^ and B^.^), which have n_1_ and n_2_ number of atoms, respectively.

Line	Column	Content
1	1	‘ref’ string specifying reference energy
1	2	reference bond separation energy (in kcal/mol)
2	1	‘molc’ string specifying start of the first molecular block
2	2	unique integer identifier, 1 indicating the A^.^ fragment
2	3	the charge of the A^.^ fragment
2	4	the multiplicity of the A^.^ fragment
3, …, n_1_ + 2	1	element type
3, …, n_1_ + 2	2	X coordinates (in Å)
3, …, n_1_ + 2	3	Y coordinates (in Å)
3, …, n_1_ + 2	4	Z coordinates (in Å)
n_1_ + 3	1	‘end’ string specifying end of the first molecular block
n_1_ + 4	1	‘molc’ string specifying start of the second molecular block
n_1_ + 4	2	unique integer identifier, 1 indicating B^.^ fragment
n_1_ + 4	3	the charge of the B^.^ fragment
n_1_ + 4	4	the multiplicity of the B^.^ fragment
n_1_ + 5, …, n_1_ + n_2_ + 4	1	element type
n_1_ + 5, …, n_1_ + n_2_ + 4	2	X coordinates (in Å)
n_1_ + 5, …, n_1_ + n_2_ + 4	3	Y coordinates (in Å)
n_1_ + 5, …, n_1_ + n_2_ + 4	4	Z coordinates (in Å)
n_1_ + n_2_ + 5	1	‘end’ string specifying end of the second molecular block
n_1_ + n_2_ + 6	1	‘molc’ string specifying start of the third molecular block
n_1_ + n_2_ + 6	2	unique integer identifier, -1 indicating the A-B parent molecule
n_1_ + n_2_ + 6	3	the charge of the A-B parent molecule
n_1_ + n_2_ + 6	4	the multiplicity of the A-B parent molecule
n_1_ + n_2_ + 7, …, n_1_ + n_2_ + N + 6	1	element type
n_1_ + n_2_ + 7…, n_1_ + n_2_ + N + 6	2	X coordinates (in Å)
n_1_ + n_2_ + 7, …, n_1_ + n_2_ + N + 6	3	Y coordinates (in Å)
n_1_ + n_2_ + 7, …, n_1_ + n_2_ + N + 6	4	Z coordinates (in Å)
n_1_ + n_2_ + N + 7	1	‘end’ string specifying end of the third molecular block

### File format

For each molecule, the reference bond separation energy and the atomic coordinates are stored in a file named *MoleculeName.db*. The Cartesian coordinates of the atoms are stored in files called *MoleculeName_AB.xyz*, *MoleculeName_A.xyz*, and *MoleculeName_B.xyz*, where *AB* represents the parent molecule, *A* represents the first radical fragment, and *B* represents the second radical fragment.

The DB format file contains a header line specifying the reference energy value (in kcal/mol) followed by three ‘*molc’* (short for molecule) blocks containing a unique integer identifier, charge, multiplicity, and the atomic coordinates (in Å) of the parent molecule and its corresponding radical fragments. The XYZ format file contains a header line defining the number of atoms N, a comment line containing the charge and multiplicity, and N lines with each containing element type and X, Y, Z coordinates (in Å). The *BSE49_Existing.org* and *BSE49_Hypothetical.org* files are special-character separated plain-text files (where the special character is ‘|’) containing multiple lines and eight columns. The columns are: (i) dataset name of the model system, (ii) unique integer identifier 1 indicating the A^.^ fragment, (iii) geometry filename of the A^.^ fragment, (iv) unique integer identifier 1 indicating the B^.^ fragment, (v) geometry filename of the B^.^ fragment, (vi) unique integer identifier -1 indicating the A-B model system, (vii) geometry filename of the A-B model system, and (viii) computational reference bond separation energy (in kcal/mol).

## Technical Validation

For the generation of reference data, the reliable (RO)CBS-QB3 method was chosen for all the model systems considered in the BSE49 dataset. The (RO)CBS-QB3 method has been widely used in literature in recent years^69–90^. The developers of the (RO)CBS-QB3 method reported that it predicts heats of formation at 298 K with a mean absolute deviation (MAD) from the experiment of 0.91 kcal/mol^33^. For bond dissociation enthalpies of eleven molecules with chemical structures typically found in amino acid sidechains, peptide termini, and peptide backbones, Moore *et al*. reported an MAD of 1.72 kcal/mol from the experimental values^8^. For small lignin model molecules, the CBS-QB3 approach was shown to yield bond dissociation enthalpies within 2.99 kcal/mol from experimental values^34^. (RO)CBS-QB3 has been used as a reference method for benchmarking various density functional theory methods to estimate bond dissociation enthalpies in a different study on small lignin model systems^23^. Hudzik and co-workers utilized the CBS-QB3 composite method to study the C-H bond separation energies of a few alkane molecules and reported a good agreement with literature values^35^. The (RO)CBS-QB3 has also been used for the prediction of bond dissociation enthalpies in a previous work by Menon *et al*.^36^ The MAD of (RO)CBS-QB3 was reported to be only 0.60 kcal/mol from the experiment and was suggested as being a reliable and efficient procedure for calculating bond separation energies in comparison to the other composite methods tested. In another work, bond dissociation enthalpies of 200 molecules were calculated using an earlier version of this work’s composite method, CBS-Q^37^. It was shown that the results of the CBS-Q composite procedure predicted bond dissociation enthalpies to within 2.39 kcal/mol of the reported experimental values. Collectively, these results support the selection of (RO)CBS-QB3 as a practical and accurate method for the generation of reference data in this work. Note that the reference bond separation energies reported in this work are non-relativistic (RO)CBS-QB3 energies without zero-point energy corrections. This makes the reference data suitable to support the development of low-cost computational chemistry methods like those described in references^28–30,38–40^.

## Data Availability

Throughout this work, the *Gaussian* software package was used for geometry optimizations, frequency calculations, and composite (RO)CBS-QB3 calculations. The *Gaussian* software package can be purchased from Gaussian Inc. (http://gaussian.com/) under a commercial license. *CSD conformer generator* was used for conformer generation. The *CSD conformer generator* can be purchased under a commercial license from https://www.ccdc.cam.ac.uk/solutions/csd-enterprise/applications/conformer-generator/. *Fullmonte* software package was also used along with *MOPAC16* (PM6-DH2 method). *Fullmonte* software package can be downloaded free-of-cost from https://github.com/bobbypaton/FullMonte. Whereas *MOPAC16* software package can be installed after acquiring a free license from http://openmopac.net/. The *Avogadro* molecular editor and visualizer is an open-source program available at https://avogadro.cc/.
